# Application of the MSAP Technique to Evaluate Epigenetic Changes in Plant Conservation

**DOI:** 10.3390/ijms21207459

**Published:** 2020-10-10

**Authors:** María Elena González-Benito, Miguel Ángel Ibáñez, Michela Pirredda, Sara Mira, Carmen Martín

**Affiliations:** 1 Departamento de Biotecnología-Biología Vegetal, Escuela Técnica Superior de Ingeniería Agronómica, Alimentaria y de Biosistemas, Universidad Politécnica de Madrid, Av. Puerta de Hierro, nº 2–4, 28040 Madrid, Spain; me.gonzalezbenito@upm.es (M.E.G.-B.); michela.pirredda@upm.es (M.P.); sara.mira@upm.es (S.M.); 2 Departamento de Economía Agraria, Estadística y Gestión de Empresas, Escuela Técnica Superior de Ingeniería Agronómica, Alimentaria y de Biosistemas, Universidad Politécnica de Madrid, Av. Puerta de Hierro, nº 2–4, 28040 Madrid, Spain; miguel.ibanez@upm.es

**Keywords:** cryopreservation, DNA methylation, multinomial model, plant tissue culture, seed storage

## Abstract

Epigenetic variation, and particularly DNA methylation, is involved in plasticity and responses to changes in the environment. Conservation biology studies have focused on the measurement of this variation to establish demographic parameters, diversity levels and population structure to design the appropriate conservation strategies. However, in ex situ conservation approaches, the main objective is to guarantee the characteristics of the conserved material (phenotype and epi-genetic). We review the use of the Methylation Sensitive Amplified Polymorphism (MSAP) technique to detect changes in the DNA methylation patterns of plant material conserved by the main ex situ plant conservation methods: seed banks, in vitro slow growth and cryopreservation. Comparison of DNA methylation patterns before and after conservation is a useful tool to check the fidelity of the regenerated plants, and, at the same time, may be related with other genetic variations that might appear during the conservation process (i.e., somaclonal variation). Analyses of MSAP profiles can be useful in the management of ex situ plant conservation but differs in the approach used in the in situ conservation. Likewise, an easy-to-use methodology is necessary for a rapid interpretation of data, in order to be readily implemented by conservation managers.

## 1. Epigenetic Variation in Ex Situ Plant Conservation: The Role of DNA Methylation Changes

Human activity in recent centuries, and particularly in recent decades, has led to overexploitation and a significant degradation of habitats, with a consequent loss of natural populations and even species. More recently, pollution and climate change have contributed to biodiversity loss [1,2]. This genetic erosion has also affected crop genetic resources due to modern agricultural practices and the introduction of new varieties, with higher yield, which have displaced traditional landraces [3]. From the 1950s, projects on germplasm conservation have been developed to guarantee biodiversity and stop genetic erosion. 

The most appropriate method to conserve whole ecosystems and their biodiversity is in situ conservation, i.e., in their natural habitat. However, this approach is not always possible, and in these cases ex situ conservation (the conservation of individuals outside their natural habitats) is the best option [2]. The most frequent plant ex situ conservation method is seed banking, mainly by maintaining orthodox seed samples at low temperature and water content. Orthodox seeds are characterized by their ability to tolerate desiccation (generally at 10% water content or lower) and to retain their viability during long-term storage in the dry state and at low temperature (generally −20 °C), reaching a glassy state during which their cellular activities and metabolism are extremely reduced [4,5]. This method can guarantee the biodiversity conservation of a high number of species in the long term and at a low cost. However, this technique is not feasible for those species with recalcitrant seeds (not tolerant to reduction of their water content and to low temperature), with asexual reproduction (e.g., hybrid species) or with high heterozygosity [6]. For these types of germplasm, in vitro conservation (slow growth) techniques and cryopreservation are successful options. Besides, another ex situ conservation procedure for plant material difficult to preserve is field collection, which has been mainly used for crop species. However, this approach requires large areas of land, is labor intensive and plants are exposed to environment changes and plagues [7].

The main target of biological conservation is to retain high levels of biodiversity. In the case of ex situ conservation, samples must represent the diversity of the natural population and of the species to be maintained. In the last years, conservation biology studies have focused on the measurement of this variation to establish demographic parameters, diversity levels and population structure in order to design the appropriate conservation strategies [8]. 

Ex situ conservation must face a double role; on the one hand, it must be representative of the diversity of the population of origin, and at the same time it must ensure the maintenance of the characteristics of the conserved genotypes. Although these techniques contribute to plant biodiversity maintenance, they present some problems that need to be solved in order to improve conservation efforts [3]. Seed banks, slow-growth and cryopreservation are the main ex situ conservation techniques that can guarantee a higher control of the samples compared to field collections. Environmental conditions for these approaches may cause an important stress to the conserved plant material. Low temperature is a common factor for these techniques, which could be from a slight reduction in the case of in vitro slow-growth (5–10 °C), to a severe reduction (near −180 °C) in the case of cryopreservation. Additionally, water content reduction is usually applied in seed banking and cryopreservation; light intensity reduction besides starvation is used in most of the slow-growth protocols.

Species can respond to new environmental situations through molecular and phenotypic changes [9]; similarly, individuals under ex vitro conservation conditions can undergo modifications to face the new conditions. In the last few years, many studies have focused on the potential role of epigenetic mechanisms in the short and long-term adaptation of species to the changing environment [10,11]. Epigenetic changes are related to changes in the genome (histone modifications, DNA methylation and siRNA), without affecting the DNA sequence. 

Among the different epigenetic mechanisms, DNA methylation, and particularly cytosine methylation is the most studied one in plants. A methyl group is transferred to a cytosine residue, forming C5-methylcytosine (5-^m^C) [12]. The enzymes that catalyze this reaction are known as DNA Methyltransferases (DNMTs), and they have primarily two general classes of enzymatic activities: *de novo* methylation and maintenance of methylation. *De novo* methyltransferases newly methylate cytosines and are mainly expressed in early embryo development. Maintenance methyltransferases act throughout the life of the organism to maintain the methylation pattern that has been established by the *de novo* methyltransferases. In plants, *de novo* methylation is carried out by Domains Rearranged Methyltransferase 2 (DRM2), a DNMT3 homolog, while maintenance methylation is catalyzed by three different processes: CG methylation by DNA methyltransferase 1 (MET1), the plant homolog of DNMT1; CHG methylation by Chromomethylase 2 (CMT2) and CMT3, plant specific DNA methyltransferases; and asymmetric CHH methylation through persistent *de novo* methylation by CMT2 and RNA-directed DNA methylation (RdDM) [13,14]. However, the pathways controlling the establishment and maintenance of DNA methylation in plants, as well as those involved in the removal of DNA methylation, are less characterized than in mammals [15].

The importance of cytosine methylation relies on the fact that it has been associated with numerous biological processes, such as genomic imprinting, transcriptional regulation of genes and transposable elements and gene silencing [13,16,17,18]. Besides, DNA methylation is considered sensitive to the environment and is involved in the plasticity and adaptative responses to changing environments [19]. Modifications of DNA methylation patterns can appear as a response of changing environments, producing “environmentally induced phenotype variation”, but may also arise spontaneously as “stochastic phenotype variation” [19,20]. Although epigenetic modifications can be reset between generations [21], some of them, especially those involving DNA methylation, may not be reset, resulting in a transgenerational stability of these markers [11]. In addition, the stability of epimutations over generations is expected to be higher in plants than in animals [22]. 

All these considerations make ex situ conservation an especially sensitive scenario in which it is important to control the state of epigenetic markers such as DNA methylation. The stressful conservation conditions may induce epigenetic changes to face new environmental situations. However, this mechanism, that in natural populations acts as an adaptive tool, may cause changes that could affect the phenotype, which would endanger the maintenance of the characteristics of the conserved plants. In the conservation context, epigenetic changes take on a greater dimension if we consider that many studies relate them with genetic mutations [23]. Jiang et al. [24] found higher frequency of mutations and epimutations (changes in cytosine methylation status) in *Arabidopsis thaliana* under salinity stress. However, although authors reported a considerable increase of both types of variation, they did not explain a possible connection between them. 

An additional problem arises when tissue culture (based on clonal propagation) is used for conservation, since epigenetic reprogramming mechanisms that are associated with meiosis can be bypassed in asexual reproduction, which could promote the build-up of epigenetic variation in vegetatively propagated plants [25].

## 2. Methylation Sensitive Amplified Polymorphism (MSAP) Technique among Other Techniques to Detect DNA Methylation Changes

There are numerous available procedures to screen DNA methylation, that have been thoroughly described. The most common techniques for analyzing DNA methylation are those based on methylation-sensitive restriction enzymes or on bisulfite modification [26].

Bisulfite sequencing is one of the main techniques used for analyzing methylation of DNA due to its high definition, since it produces results with single-nucleotide resolution [27]. Genomic DNA is treated with sodium bisulfite, provoking the deamination of unmethylated cytosines, which results in their conversion to uracil while methylated cytosines remain stable. Subsequently, bisulfite-treated DNA is amplified by PCR using specific primers, and uracil residues are replaced by thymine. Amplification fragments are sequenced allowing the identification of methylated cytosines. This technique is also suitable for genome-wide analyses. Bisulfite treatment generates high resolution outcomes, but its high cost, time and intensive labor are some of its main limitations, especially in genome-wide analyses of DNA methylation [27]. Additionally, there is a risk of incomplete conversion of unmethylated cytosines to uracil [28] or DNA degradation via depurination because of the high temperatures and bisulfite concentrations used in the process [29].

Other techniques are based on the use of restriction enzymes with different sensitivity to methylation such as the combination of *Msp*I and *Hpa*II isoschizomers. These restriction enzymes are used in Methylation Sensitive Amplified Polymorphism (MSAP) technique and differentially cleave their recognition site 5′-CCGG-3′ based on methylation differences of cytosine residues.

The MSAP approach was first described by Reyna-Lopez et al. [30] in a study on fungi and later modified for its use in plant species by Xiong et al. [31]. Ever since, the method has been adopted in more than 100 publications, focusing mainly on developmental biology (e.g., [32,33]), hybridization and polyploidization (e.g., [34]), plant breeding (e.g., [35]) and plant response under stress conditions [36,37,38]. More recently MSAP analyses also became an important tool to answer questions in the emerging field of “ecological epigenetics”, studying epigenetic processes in an ecological context [19].

MSAP is fundamentally a modification of the Amplified Fragment Length Polymorphism (AFLP) method based on the digestion of genomic DNA with methylation-sensitive restriction endonucleases followed by the amplification of digested fragments. In the MSAP protocol, the extracted genomic DNA is divided into two aliquots, each digested with *Eco*RI, which recognizes the GAATTC target site and is thought to be negligibly influenced by DNA cytosine methylation (“indifferent cutter”). The aliquots are then digested with the methylation-sensitive *Msp*I or *Hpa*II isoschizomers, respectively (“methyl-sensitive cutter”), which recognize the same restriction site (CCGG) but show differential sensitivity to cytosine methylation. The DNA samples digested with *Eco*RI and *Msp*I or with *Eco*RI and *Hpa*II are ligated to two dsDNA adapters compatible with *Eco*RI and *Msp*I/*Hpa*II-generated ends. Subsequently, ligated fragments are pre-amplified using non-selective or pre-selective primers complementary to the adapters followed by amplification with a pair of selective primers (these are one- to three-base extended variants of non-selective or pre-selective primers at 3′ ends). Such amplification produces a reduced population of fragments that are separated in order to compare the respective band patterns [39,40]. 

It is important to mention that although *Hpa*II and *Msp*I recognize the same motif (5′-CCGG-3′), literature is inconsistent regarding their cleaving activity in different methylation contexts (e.g., [41,42,43]). According to Schulz et al. [39], and following the methylation sensitivity criteria of the restriction enzyme database REBASE [44], *Hpa*II only recognizes sites that are hemi-methylated at the external cytosine (^m^CCGG), while *Msp*I only recognizes sites being hemi- or fully methylated at the internal cytosine (C^m^CGG). None of the enzymes cut at the recognition site when it is fully methylated at the external cytosine, or hemi- or fully methylated at both, internal and external, cytosine residues. However, when there is no methylation in CCGG-sequences, both enzymes can digest [39]. For each sample there are two sets of amplification data (one from each restriction enzyme). The binary information for each fragment (present/absent) reveals its methylation status.

One of the main advantages of this technique in the plant conservation context is that MSAP allows for research on non-model systems, even if their genome is not sequenced, as the amplification of restriction fragments is independent on the availability of genome sequence information [40].

Technically, MSAP is similar to AFLP, a procedure that has been well documented over the years; both techniques require the same equipment, similar protocols and expertise. Furthermore, this method is cost-effective, with minimal start-up and ease to scale-up, as the same reagents can be used on multiple taxa [45]. In addition, it generates powerful data to detect differences among populations or treatments, as it can screen a large number of individuals at multiple loci concurrently. These characteristics make this technique very versatile, resulting effective in a wide variety of studies focused on different biology aspects, such as ecology, plasticity, preservation or evolution.

On the other hand, the main shortcoming of MSAP is that it screens anonymous loci [45]: it cannot specify the region or gene influenced by methylation because the sequence adjacent to each locus remains unknown. A candidate solution for this drawback could be the extraction and sequencing of the fragments obtained and the database search for homologous sequences to those fragments (BLAST). It is important to mention that the extraction of MSAP bands is extremely laborious, because of the small band size and the large number of bands obtained. In the analyses of MSAP results, it is complicated to establish a relationship between methylation and phenotype, as there is not always an explicit connection between DNA methylation and gene-expression. This issue could possibly be assessed by performing association mapping to link phenotype to epigenetic states at particular loci [46]. Likewise, it must be considered that MSAP results in a dominant banding pattern and, therefore, it is not possible to distinguish heterozygote epigenotypes. There is a further technical shortcoming regarding the MSAP procedure: the banding pattern observed when both *Msp*I and *Hpa*II fail to cut. Such conditions can be generated by both genetic (point mutation to the restriction site, or changes to adjacent restriction sites) and epigenetic (hypermethylation, methylation of all cytosines in the restriction site) causes. Thus, some methylated states may remain undetected.

Among other techniques based on methylation-sensitive restriction enzymes, methylation-sensitive amplified fragment length polymorphism (metAFLP) can be mentioned. This technique is also a modification of the AFLP technique, but it uses different endonucleases to those used in MSAP. *Acc*65I and *Kpn*I are isoschizomers, which differ in their sensitivity to template methylation, and, together with *Mse*I are used for the initial digestion of genomic DNA [47]. 

## 3. Seed Conservation

Ex situ conserved seeds, even if the most optimal storage conditions are used, are subjected to ageing and this results in the loss of valuable genetic diversity. To avoid genetic loss over time, seed accessions are regenerated; nevertheless, this represents an expensive procedure and could lead to the genetic drift of the accession by selection, contamination, presence of mutations or human error [48]. There are over four million seed accessions worldwide in germplasm banks nowadays [49], approximately two thirds in long term storage. The genetic and epigenetic stability of all that stored biodiversity is, therefore, of the upmost importance.

Seed ageing has been described as the loss of seed quality overtime. Several physiological and biochemical changes have been associated with seed ageing: reactive oxygen species (ROS) accumulation, lipid peroxidation, membrane phospholipids loss, decrease in the activity of antioxidant enzymes [50,51], impaired protein synthesis, protein inactivation, changes in enzyme activities, protein hydrolysis, and post-translational modifications [48,52], among others. ROS interact with cellular biomolecules, and can cause serious oxidative damage to proteins, nucleic acids and lipids [53,54]. Furthermore, some of the by-products of lipid peroxidation, such as the aldehydes malondialdehyde (MDA) and 4-hydroxy-2-nonenal (4-HNE), are highly reactive [55]. Both molecules have been shown to interact with proteins (leading to loss of function) and DNA (leading to mutations) or inhibit DNA and protein synthesis [53,56]. Besides, epigenetic regulation, in particular DNA methylation, has been proposed as a possible indicator of seed ageing [57] as it has been related to viability loss during seed storage as the following reported studies show. 

MSAP has been scarcely used to reveal the methylation status of stored seeds (Table 1). Pirredda et al. [58] studied non-stored and stored rye (*Secale cereal* L.) seeds at different stages of ageing, as well as the seedlings obtained from them. Seeds were stored at 35 °C and 15% water content fresh weight basis (wc. fwb.), under vacuum or air atmosphere. DNA methylation-related changes (15–30% both *de novo* methylation and demethylation) were detected in the stored seeds compared to control seeds. These variations were not associated with storage time, even when germination was significantly reduced with time (25% and 80% of germination reduction after 13 and 29 days, respectively). However, DNA methylation-related changes significantly increased with storage time in the seedlings obtained from the stored seeds: from 13% after 13 days to 23–27% after 29 days. In this study, the effect of storage conditions (time and atmosphere) on the methylation status in stored seeds and seedlings was analyzed by a multinomial logistic regression model. In *Mentha aquatica* L. [59], the DNA methylation changes detected increased from 8% in stored seeds (compared to control seeds) to 16% in the seedlings produced from them, compared to those obtained from control seeds. 

Despite the scarcity of studies using MSAP technique, methylation status in stored or desiccated seeds and, in some cases also in the derived seedlings, has been studied with other methods, such as two-dimensional thin-layer chromatography (TLC). In TLC, ^m^C and other nucleotides are labelled with [^32^P] ATP and T4 polynucleotide kinase; the amount of global ^m^C is calculated as a spot intensity ratio [60]. The TLC technique has been used combined with MSAP in vernalization studies [61]. By means of TLC, it was observed that in the common pear (*Pyrus communis* L.) the global level of DNA methylation decreased in seeds with very low water content (2.8% wc. fwb.) compared to 8.8% wc. (control seeds); desiccation also produced a slight germination decrease [62]. Similarly, 3-month old seedlings obtained from dried seeds showed lower DNA methylation than seedling from control seeds. In another work of the same group, DNA methylation of *Acer platanoides* L. seeds (orthodox), increased when they were desiccated from 51% wc. fwb. to 15% [63]. However, when further desiccation was imposed (9–6% wc.) the methylation level decreased, together with germination and seedling emergence, especially in the seed lots collected at higher moisture content (51% *vs.* 21% wc. fwb.). Even though *P. communis* and *A. platanoides* seeds are classified as orthodox, they seem to differ in their tolerance to extreme desiccation. Furthermore, these authors also compared the methylation levels of embryonic axis and cotyledons from two species of the same genus, but with different storage behavior. In embryonic axes of both *A. platanoides* (orthodox) and *A. pseudoplatanus* L. (recalcitrant) lower methylation DNA levels were observed as the water content decreased; however, this effect was only found in the cotyledons of *A. pseudoplatanus* [64]. These results indicate that desiccation-induced changes in total DNA methylation are both tissue- and seed category-specific. Moreover, the methylation levels of 3-month old seedlings derived from seeds at different water contents were similar among them, except for *A. platanoides* seedlings from severely desiccated seeds (3.5% wc.), despite the germination decrease observed in all desiccated samples. 

Orthodox seeds can also be stored in liquid nitrogen without the need of pretreatments. The percentage of methylated DNA has been studied in cryopreserved maize (*Zea mays* L.) kernels and seedlings generated from them [65]. DNA methylation was determined by MSAP although no statistical analysis was performed. The percentage of DNA methylation was similar in cryopreserved and non-cryopreserved kernels (72% *vs*. 65%). In 5-day old seedlings, shoots derived from non-cryopreserved seeds showed higher methylation levels than those from cryopreserved seeds, while the opposite was observed in roots. As seedling growth proceeded (9-day old seedlings), DNA methylation in shoots from cryopreserved seeds increased, while it decreased in seedlings from non-cryopreserved kernels. Those differences in the methylation status of seedlings could be related to a slight growth delay observed in those obtained from cryopreserved seeds.

By means of metAFLP, no differences were found in the methylation level of 2-week old rye plants derived from seeds stored for 25 years either under conventional seed banking or cryopreserved, although cryopreserved seeds showed higher percentage of normal germination [66]. 

## 4. In Vitro Plant Conservation

The main strategy for in vitro conservation is “slow growth”, which is achieved by modifying environmental conditions and/or medium composition with the aim of limiting plant metabolism and growth. This approach is mainly used for short- or medium-term conservation. The growth limitation allows prolonging subculture intervals without significantly affecting the viability of the explants [67].

Temperature reduction is the most widely applied modification, which can be combined with a decrease in light availability (low radiation or short photoperiod), or even darkness. Another common limitation is the reduction of macro- and micro-nutrients of the medium, sometimes combined with a decrease in sucrose concentration. Modifications of the medium osmotic potential are also used to reduce the water availability (e.g., addition of mannitol or sorbitol). The use of plant growth retardants is another strategy, although less frequent [67,68,69,70].

Slow growth has been applied in the last few years to many species, mainly for medium-term conservation, including diverse crops [69], ornamental plants [70] and endangered species [71].

Since the stressful conditions imposed by tissue culture procedures, and their implications on the epi-genetic stability of cultured material, are well known [72,73], many studies using molecular markers have been carried out to examine the genetic stability of in vitro conserved cultures [74,75,76]. However, the number of studies focused on the DNA methylation-related stability of slow-growth cultures is scarce (Table 1).

The first study on DNA methylation of plants recovered from slow-growth was performed by Harding [77] in potato (*Solanum tuberosum* L.) using a technique based on the use of isoschizomers *Hpa*II/*Msp*I and other restriction enzymes, but different to MSAP. In this work, morphological changes and hypermethylation of genomic DNA were detected in plants conserved in a medium supplemented with mannitol. The author attributed methylation changes to a possible adaptive response to high stress osmotic conditions.

The MSAP technique was used by Hao and Deng [78] in apple (*Malus pumila* Mill.) shoot tips conserved for one year at 4 °C and a photoperiod of 12 h, with a medium supplemented with 2% mannitol. Using AFLP markers no genetic variation was detected between the conserved samples and the shoot prior to storage (control). However, 6 out of 389 analyzed markers changed in the MSAP study. These changes were not attributed by authors to *de novo* methylation, nor to demethylation, but to changes from hemi-methylation to full methylation status. The variation of the DNA methylation status was considered a response of the plants to different stresses associated with in vitro conservation conditions. Despite the significant variation detected, the authors justified the use of this conservation technique as an advantage over field collections. 

Not only shoots are subjected to in vitro conservation, other explants, such as callus, have been stored for medium-term, usually associated to breeding programs, as in the case of *Citrus* callus [79]. The previously mentioned research group, working with callus of grapefruit, analyzed the epi-genetic stability of callus stored in slow-growth conditions for one year (Table 1). Genetic stability was assessed by Random Amplified Polymorphic DNA (RAPD) markers and ploidy level, and no significant differences were found. However, the MSAP analysis revealed one variation (among 308 markers analyzed) attributed to a demethylation event. 

In studies on the use of slow growth in hop (*Humulus lupulus* L.) germplasm collections, Peredo et al. [80,81] found changes in the DNA methylation status of the in vitro plants conserved for one year at 4 °C and 12 h photoperiod (Table 1) when compared with greenhouse control plants. The response of the three genotypes analyzed varied, but changes were detected (11.2–18.3% of the analyzed markers) in all of them, corresponding mainly to demethylation events (4–11% of the detected changes, depending on the genotype). As in the previous mentioned works, genetic analysis was done using RAPD and AFLP markers, and similarly to those studies no genetic variation was detected. For these authors, the explanation of the DNA methylation changes observed laid on the procedures used in the in vitro culture, while conservation conditions per se had a minor effect. This conclusion was drawn from the comparison with cryopreservation results, which also have an in vitro common protocol (see next section).

Slow growth storage has been used more recently in the conservation of synthetic seeds of diverse species [82,83,84], applying a reduction in the conservation temperature. Although in some cases the genetic stability was assessed using molecular markers or flow cytometry [82,84], the DNA methylation status was not analyzed.

The lack of studies about the DNA methylation-related status of plants from slow growth storage does not mean that this technique is not being applied nowadays, as there are over fifty thousand accessions stored in vitro [49]. Although the technique is widely employed in germplasm banks and conservation institutions, analyses are not frequent enough. Furthermore, when stability studies have been carried out, they have focused primarily on genetic stability, as for example the use of Simple Sequence Repeat (SSR) markers in the analysis of conserved artichoke (*Cynara cardunculus* L.) [85], Inter Simple Sequence Repeat (ISSR) markers in tomato (*Solanum lycopersicum* L.) [86] and RAPD together with flow cytometry in slow growth of *Taraxacum* [87].

The scarce works published in this area showed significant changes in the DNA methylation status of the conserved plants although genetic changes have not been detected. However, methylation changes can produce phenotypic variations affecting the true-to-type identity of the conserved material. Likewise, it is well known that these changes may be involved in the activation of transposable elements and may also affect cytogenetic stability [88]. 

Harding [77] and Peredo et al. [80,81] attributed the DNA methylation changes detected in slow growth to the in vitro culture procedures. Studies of the effect of tissue culture on the DNA methylation stability have detected significant changes, as for example the work of Gimenez et al. [89], on in vitro propagated garlic, a species usually conserved in germplasm banks through slow growth storage. These authors, using MSAP, detected changes, mainly demethylations, in plants under prolonged in vitro culture. These findings support the need to evaluate the DNA methylation status of the conserved material, mainly considering that the core objective of this procedure is to maintain the integrity and functionality of samples [85]. Techniques as MSAP may be a useful tool to analyze plants obtained from slow growth conservation. In addition, a deeper study on the effect of conservation conditions on stability (temperature, light, added substances, etc.) could result in a better development of conservation techniques in order to obtain high quality conserved plants according to integrity values. Sequential analyses have been carried out in other conservation techniques such as cryopreservation (see next section), and similar studies could help to understand the slow growth process and its implications in DNA methylation.

## 5. Cryopreservation

Plant cryopreservation allows for the long-term storage of valuable germplasm otherwise difficult to preserve. Cryopreservation is the storage of live cells, tissues or organs at temperatures below −150 °C, which ensures an extremely low metabolism, allowing long-term storage. As mentioned before, these techniques have special importance when conserving diversity of plants with recalcitrant seeds, short-lived seeds or vegetatively propagated [55,90,91,92]. Worldwide there are over 700,000 cryopreserved accessions of crop species representing 13.12% of the total number preserved in germplasm banks [49]. Besides, cryopreservation is often considered as the only effective method to prevent cell ageing and reduce the risks of culture loss caused by contamination or technical errors when preserving in vitro cultures of undifferentiated somatic plant cells [93], used as a source of phytochemicals for food and pharmaceutical industries. 

In order to avoid ice crystal formation and/or desiccation damage in cells, two main types of cryopreservation techniques have been developed [6,94]. Some are based on a controlled decrease of temperature and the use of cryoprotectants, forming extracellular ice crystals and causing the cells to dehydrate to the point where they would turn to a glass (vitrify). The second type is based on the vitrification of both extra- and intracellular solutions, by the concentration of solutions and their fast cooling, without undergoing crystallization. Among these techniques are the ones based on the use of vitrification solutions (vitrification *sensu stricto*) and those based on encapsulation-dehydration. In most cryopreservation protocols, using any of these techniques or their modifications, plant cells, tissues or organs are generally preconditioned/pretreated by, for example, in vitro culture on medium with high sucrose concentration or containing other cryoprotective substances, or by incubation at low temperature. The relationship between cryopreservation and in vitro culture is, therefore, very close as often the plant vegetative material used in cryopreservation is obtained and recovered in in vitro culture, and pretreatments are applied also in vitro. The papers reviewed in this section refer to studies on cryopreservation of in vitro plant material. 

The treatments imposed on cells to avoid intracellular ice formation or extreme dehydration produce stresses at the cellular level that, although they may not lead to cellular death, could produce alterations in biomolecules [55]. Damages to cells have been related to the toxicity of cryoprotectants, cell membrane integrity alteration, mitochondria disruption, or oxidative stress [55,95]. Oxidative stress constitutes a major component of cryo-injury, caused primarily by ROS [95,96]. Each of the steps in the cryopreservation protocol presents the possibility of oxidative damage, due to physical damage (excision) and the osmotic stress involved in the process, as many studies have shown [97]. 

As has been mentioned before (see Section 4), the stressful conditions of in vitro culture could account for the epigenetic changes observed after cryopreservation [98]. Nevertheless, changes in DNA cytosine methyltransferase expression and changes in histone acetylation or methylation have been reported after cryopreservation of bovine embryos and mouse and pig oocytes, respectively [99]. 

Although a considerable amount of literature has been published on plant genetic stability after cryopreservation [2,100,101,102], the effect that this process has on epigenetic stability has been scarcely approached. MSAP is one of the techniques most widely used for DNA methylation-related studies of plant material after cryopreservation. Other employed methods are amplified DNA methylation polymorphism (AMP [103]) and metAFLP [104]. Johnston et al. [105] studied total DNA methylation by high-performance liquid chromatography (HPLC). 

Most of the studies in which MSAP were used to evaluate DNA methylation changes after cryopreservation showed that demethylation events were the most frequent, when compared to the non-cryopreserved control plant material (Table 1). 

Hao et al. [106] studied the genetic and DNA methylation stability of apple (*M. pumila*) in vitro shoot tips after cryopreservation by encapsulation-dehydration. While no changes were observed in genetic markers (AFLP), MSAP showed five demethylation events in cryopreserved shoot tips when compared to non-cryopreserved ones out of the 380 bands observed. The authors hypothesized that the change in the DNA methylation status could have been related to the observed enhancement of root capacity after cryopreservation, as DNA demethylation/methylation in plants play an important role in regulating plant development and organ or tissue differentiation [107]. Hao et al. [108] also found similar results after the cryopreservation of strawberry (*Fragaria vesca* L.) shoot apices, again by encapsulation-dehydration, although this time the frequency of demethylation events was lower: 1 out of 314 bands. An increase in demethylation events after cryopreservation compared to in vitro control plant material has also been reported using vitrification-based protocols. *Citrus* callus showed 1 de novo methylation and 3 demethylation sites, out of approximately 358 markers [109]. 

Potato shoot tips, derived form in vitro plants, were cryopreserved by the DMSO-droplet method and stored for 7 years, while another group of in vitro plants were maintained for the same period with periodical subculture [110]. The methylation status of cryopreservation-derived shoots and shoots maintained in vitro were compared in three random biological samples selected from both groups. The changes in the methylation events were low (0.9%), most of them being demethylation events (0.6%). However, there were cases in which the biological repetitions for the same treatment differed; most of the changes (3.4%) were demethylation events in particular cryopreserved samples [110].

Zhang et al. [111] compared the DNA methylation status of kiwi (*Actinidia chinensis* Planch.) plants originated from cryopreserved (by vitrification) apices to those from in vitro multiplication. Plants were studied at two developmental stages after recovery: after 8 weeks of in vitro culture after cryopreservation or after further 3 months acclimation in the greenhouse. In the cryopreserved derived plants, more changes (compared to the in vitro counterparts) were observed in in vitro than in acclimatized plants, which could indicate transient changes: 52 methylation changes vs. 7, out of 718–701 bands. In the in vitro grown plants 30 of the 52 changes were demethylation events and 22 de novo methylation events. 

Adu-Gyamfi et al. [98] compared, by MSAP analysis, cocoa (*Theobroma cacao* L.) somatic embryos, multiplied by in vitro culture or cryopreserved and subsequently multiplied, with the tree from which the starting material for the embryogenesis was obtained. They did not consider if the methylation changes obtained were demethylation or *de novo* methylation. However, they found an increase in DNA methylation-related variability in all in vitro and cryopreserved samples, especially in the latter. The DNA methylation-related distance (calculated using Analysis of Molecular Variance inferred from the analysis of epiloci) to the donor plant of the cryopreserved and subsequently cultured embryos was 0.65 and that of the in vitro maintained embryos 0.48. The authors had found in previous works phenotypic variability in cryopreserved cocoa somatic embryos but little genetic instability; therefore, they hypothesized that those phenotypic variations may be due to DNA methylation changes. 

The DNA methylation-related status of three hop cultivars after in vitro cold storage or cryopreservation was compared to potted greenhouse-grown plants [80]. The cold stored shoots were initiated in vitro and stored at 4 °C for a year. The shoots originated from slow-cooling cryopreserved apices, stored in liquid nitrogen for three years, were recovered and grown in vitro for further 4 months. Both treatments shared a common step of 1–2 weeks of cold acclimation at −1 °C and 16-h dark/22 °C 8-h light. The percentages of methylation events changes were 35.7% and 36.73%, respectively, for cold- and cryo-stored plants; 63.61% of those changes were shared by both treatments. For both treatments, approximately 47% of changes were due to demethylation. The high proportion of common changes could be explained by the in vitro growth of both types of plant material as they were compared to potted plants, or by the common cold acclimation step.

**Figure 1:**
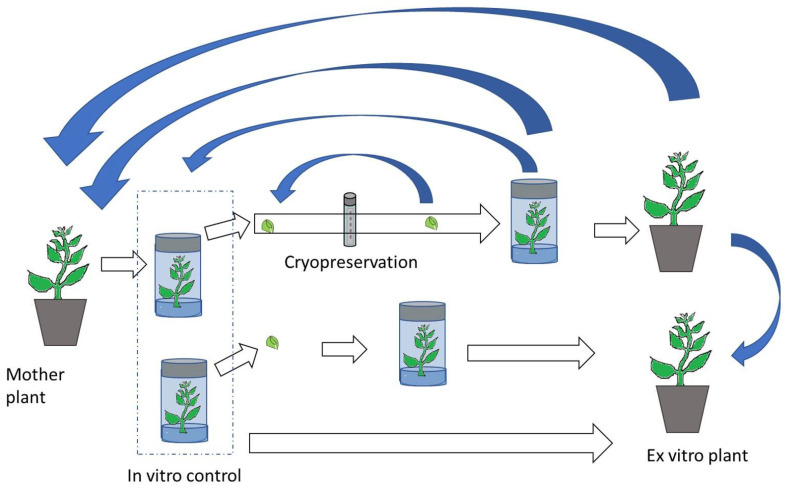
Possible comparisons established after in vitro or cryopreservation protocols for DNA methylation studies to check the fidelity of conserved plants. White arrows: culture process. Blue arrows: comparisons between stages.

The discussion of the methylation changes observed after cryopreservation is somehow complex due to the different developmental stages at which the DNA of the treated and the control samples is extracted (Figure 1). DNA methylation level varies among different plant tissues and also at different developmental stages [107]. The comparison of plant material at different developmental/physiological stages will generate differences in the methylation pattern without discerning if those changes are due to the treatments applied or to the plant stage.

**Figure 2:**
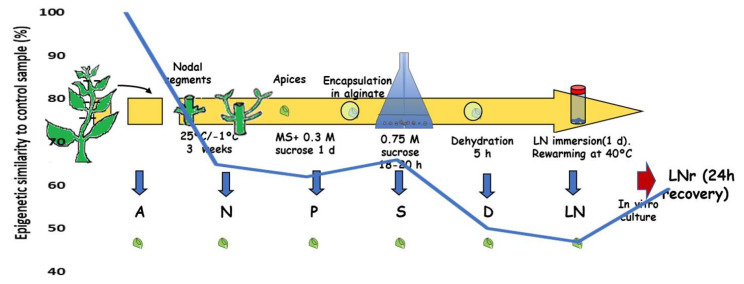
Steps of the cryopreservation protocol at which the methylation status of mint apices was studied in Ibáñez et al. [112]: control (A), cold acclimation (N), preculture in sucrose (P), alginate beads in sucrose (S), dehydration (D), immersion in liquid nitrogen (LN), immersion in liquid nitrogen and one day recovery (LNr). Curve represents the percentage of DNA methylation similarity to the control sample.

Ibáñez et al. [112] studied the methylation changes in mint (*Mentha* × *piperita* L.) shoot apices just after each step of the cryopreservation protocol by encapsulation-dehydration, without further in vitro growth. This allowed them to determine the accumulated effects of each treatment applied. The control sample consisted of apices from in vitro cultured shoots. The percentages of methylation changes increased significantly along the protocol compared to control apices (step “A” in Figure 2): from 35% after the cold acclimation treatment (N) to 53% in apices recovered from liquid nitrogen (LN). Contrary to previous works on methylation changes after cryopreservation, the most frequent events were *de novo* methylation (59% after LN step). However, after one-day in vitro recovery (LNr), the methylation changes reverted to only a 40.8%, therefore becoming more similar to control apices.

**Table 1:** Studies of DNA methylation stability of conserved plant germplasm using Methylation Sensitive Amplified Polymorphism (MSAP) markers. Wc.: water content; fwb.: Fresh weight basis; √: genetic stability reported; NA: study not carried out; SE: somatic embryos.

Species	Studied Organ	Control	Conservation Technique	Genetic Stability	Detected DNA Methylation Variability	Ref.
Seed conservation						
*Zea mays*	Caryopsis	Non-cryopreserved caryopsis	Caryopsis stored 12% wc. fwb. and storage in liquid nitrogen for 1 year	NA	Increase in the DNA methylation percentage from 65.2% to 72.6%	[65]
*Secale cereale*	Embryo and seedlings	Embryos and seedlings from non-stored caryopsis	Caryopsis stored 35 °C and 15% wc. fwb. stored for 13 or 29 days	√ RAPD in embryos, 5% changes in seedlings	15–30% DNA methylation changes in seeds; 13–27% in seedlings	[58]
*Mentha aquatica*	Seeds and seedlings	Non-stored seeds, and seedlings from non-stored seeds	Seeds stored at 35 °C and 12% wc. for 28 days	√ RAPD in seeds; 13 % in seedlings	8% DNA methylation changes in seeds, 16% in seedlings	[59]
In vitro slow growth						
*Malus pumila cv.* Gala	In vitro shoots, from single bud	In vitro buds; state of development not stated	Half-strength medium, sucrose reduction, 2% mannitol, 4 °C, 12 h photoperiod, for 1 year	√ AFLP	6 changed markers out of 389 (changes from DNA hemi-methylation to full methylation status)	[78]
*Citrus paradise* *cv.* Red Marsh	Embryogenic callus	Embryogenic callus	Half-strength medium, sucrose reduction, 10 °C, darkness, for 1 year	√ RAPD	1 DNA demethylation marker out of 314	[79]
*Humulus lupulus*	In vitro shoot cultures	Greenhouse plants	4 °C, 12 h photoperiod, for 1 year	√ RAPD, AFLP	35.7% loci changed, of which 4–11% DNA demethylation	[80,81]
Cryopreservation						
*Malus* *pumila cv. M26*	In vitro shoots from cryopreser-ved apices, from single bud	In vitro buds; state of development not stated	Encapsulation–Dehydration	√ AFLP	5 DNA demethylation markers out of 380	[106]
*Fragaria vesca*	In vitro shoots from cryopreser-ved apices, from single bud	In vitro shoots	Encapsulation–Dehydration	√ AFLP	1 DNA demethylation markers out of 314	[108]
*Citrus*	Callus after cryopreser-vation, single cell line	Callus	Vitrification PVS2	√ RAPD	1 DNA de novo methylation, 3 DNA demethylation markers, out of 358	[109]
*Humulus lupulus*	In vitro shoot form cryopreser-ved apices	Greenhouse plants	Slow cooling	√ RAPD, AFLP	36.73% loci polymorphic, of which aprox 47% DNA demethylation	[80]
*Solanum tuberosum*	In vitro plants from cryopreser-ved shoot tips	In vitro plants	DMSO-droplet method	NA	3 DNA demethylation and 1 DNA de novo methylation markers out of 469	[110]
*Theobroma cacao*	SE (cryo + in vitro SE)	Leave from ortet tree	Vitrification	NA	DNA methylation-related distances of 0.5 (similar to those of in vitro SE)	[98]
*Mentha*x *piperita*	In vitro shoot apices after each step of the protocol	In vitro shoot apices	Encapsulation-dehydration	√ AFLP, RAPD	53% DNA methylation changes were observed (being 59% de novo methylation), which was reduced to 40.8% after one day recovery	[112]
*Actinidia* *chinensis* var. *deliciosa.*	8wk-old in vitro shoots derived from cryopreser-ved apices, and 3 mo-old ex vitro plants	Corresponding in vitro-derived samples	Droplet-vitrification	√ ISSR, AFLP	In vitro: 22 DNA de novo methylation and 30 DNA demethylation markers out of 718 Ex vitro: 6 DNA de novo methylation and one DNA demethylation marker out of 701	[111]

## 6. Statistical Methods for MSAP Analysis in Plant Conservation

A specific DNA methylation state reflects the outcome of the dynamic regulation of establishment, maintenance and removal activities: *de novo* methylation, maintenance of DNA methylation, active DNA methylation and passive DNA methylation [113,114]. These activities are catalyzed by several enzymes that act in a coordinate fashion and are activated by different mechanisms, making the level of DNA methylation in the cells dynamic and variable, and therefore affecting MSAP results analyses.

There are many factors that affect the level of methylation in CCGG sequences and that cause the number of fragments produced by the MSAP technique to vary from sample to sample. In ecological and population DNA methylation-related studies [39,115], where DNA samples are obtained from several individuals, there is variation in GC content and methylation level between those individuals. In these studies, it is important to consider that during the sampling process an additional statistical variation is generated due to the sampled individuals. In addition to the inter-individual variation in DNA methylation, there are variations in the levels of methylation between tissues of an organism because of differences in gene expression in the process of cell growth and differentiation [113].

There are many studies that relate the level of DNA methylation and abiotic and biotic factors both in plant physiology and in vitro culture [89,112,116,117]. In these cases, it is also important to consider the methylation variation caused during the experiment (stochastic variations). For this reason, it is appropriate to use multiple replicates in each experimental condition to determine if the observed differences are due to the factors investigated or we are simply detecting experimental variability.

Likewise, together with the biological variability in the methylation level of the samples, it must be taken into account that although the MSAP technique is quite reproducible, there may be technical errors that cause a fragment to be absent or present [118]. In this sense, some authors replicate independently some samples to be studied by the MSAP technique; they determine an error rate per fragment and only consider to analyze those fragments with error rates lower than a threshold value [89,119,120]. These authors report error rates ranging from 2% to 10%. In this regard, Bonin et al. [118] indicated the possibility of developing statistical models that incorporate these errors and assess their impact on the final inference.

Therefore, the variation in the measured outcome detected in MSAP analysis is the cumulative effect of all these types of variation (i.e., genetic, environmental, and experimental variation) and any additional unexplained variation. These variations must be considered in the data analysis for a proper interpretation of the results (precision of the estimates, level of significance). Although in the particular case of in vitro culture studies genotype variation is usually not considered since samples are clones from a unique genotype, the rest of the causes still apply and may affect the degree of methylation and their subsequent detection.

In population genetic studies, the statistical techniques used are mainly multivariate methods. They calculate a similarity index between the experimental conditions (Jaccard, Nei, Dice index), and with these matrices they perform principal coordinate analysis (PCoA) and hierarchical classification with the unweighted pair-group method using arithmetic average algorithm (UPGMA) aimed at estimating the DNA methylation-related dissimilarities between populations. They usually carry out resampling methods (bootstrap, permutation) to obtain the precision of the estimates they produce.

Some studies on in vitro culture related to conservation procedures (slow growth and cryopreservation) do not report how many DNA samples from each experimental condition were used to perform the MSAP analysis. In some cases, a single sample was used per experimental condition, it being sometimes a pool of several samples [111,121,122,123]. Or the fragments obtained from the different analyzed samples were counted together without considering the variability between the samples within each experimental condition. It is also frequent to find comparisons among individuals or tissues of different developmental or physiological state, with a consequent misinterpretation of the results.

In most of these studies, only a description of methylation events and/or changes in methylation between different conditions was stated [111,121,122,124]. A statistical analysis of this type of samples would only reflect the variability between fragments for the different experimental conditions but does not allow to compare it with the experimental error.

When a statistical analysis was carried out, most of the published works used the multivariate techniques proposed in the studies of population epigenetics [80,98,123]. These methods are suitable in the context of population epigenetics, where a large number of individuals within the population are measured and the interest is to quantify diversity based on differences of DNA methylation markers between populations. However, in in vitro culture studies or experimental studies on the influence of abiotic or biotic stress on DNA methylation, the main interest is to show how and to what extent experimental conditions affect methylation and demethylation processes. To this end, some authors used ANOVA and the two-sample t-test in their studies to compare the percentages of the different methylation events that can be detected with the MSAP technique [89,125]. The drawback of these analyses is that they are assuming that the methylation events follow a normal distribution, which in the case of the presence/absence of fragments, may not be correct.

Ibáñez et al. [112] developed an on-line application (Methylation Analysis Inference—MAI—application) to facilitate the statistical analysis of MSAP markers. This application uses the multinomial distribution to model the different methylation events detected with the MSAP technique. Although it can be used to analyze changes *versus* a control (unchanged, *de novo* methylation and demethylation), the approach is also valid for modeling events detected directly from MSAP markers (without a control), using the binomial distribution in the case of only two methylation events. This approach takes into account the variability between samples within each experimental condition. To achieve this, several biological replicates per experimental condition should be used, and each replication independently digested with MSAP enzymes provides a different fragment pattern in each sample. Statistical analysis separates experimental variability and determines if the differences detected could be due to changes in experimental conditions and not just a consequence of experimental variability. MAI application has been used to analyze MSAP data from cryopreserved apices [112] and stored seeds [58].

## 7. Conclusions

There are over five million accessions stored worldwide by different means, which play a crucial role in both food security and biodiversity maintenance. The different storage procedures (seed banking, in vitro and cryopreservation) impose stresses to plant cells that could cause several molecular alterations including epigenetic changes. Although still far from being a reality, it would be compelling and useful to find epigenetic changes associated to specific stresses imposed by storage conditions that could be used as biomarkers. Here, studies on DNA methylation variability occurring under storage condition have been reviewed. Demethylation events were the most frequently reported after storage. Although most of these changes are likely to be transient, some could be transferred to offspring. The level of DNA methylation changes detected in the different samples strongly differs resulting significantly high in some cases and quite low in others. That could be related to either a different capacity of different species to cope with stress imposed by storage conditions or to different storage conditions or sample stage. However, the different authors report their results in a non-homogeneous way, which makes it difficult to establish clear conclusions regarding the methylation changes occurring during ex situ conservation. In general, there are still many aspects to be clarified on the relationship between germplasm storage and DNA methylation. To this aim, MSAP is an easy-to-use technique that does not require previous knowledge of the species genome that could be used for screening DNA methylation variations occurring during storage. However, it must be considered that DNA methylation pattern not only differs between species but can also be stage- and tissue-specific. As the main objective is to maintain the genetic and functional integrity of stored samples, comparisons with non-stored plant material (control) should be performed paying attention to compare samples at similar developmental stages. Therefore, the comparison of, or example, in vitro and ex vitro plants will, undoubtedly, result in differences in the epigenetic status (Figure 1). Nevertheless, an adequate design of the sampling and a sound statistical analysis are necessary to draw clear conclusions. Thus, the number of replicates should be high enough to account for stochastic variation. The use of appropriate statistical analysis will help to discern among stochastic and treatment-induced changes and will facilitate the development of more appropriate conservation methodologies [112].

## Abbreviations

AFLP	Amplified Fragment Length Polymorphism
AMP	Amplified DNA methylation polymorphism
CMT	Chromomethylase
DNMT	DNA Methyltransferases
DRM	Domains Rearranged Methyltransferase
fwb	Fresh weight basis
HPLC	high-performance liquid chromatography
ISSR	Inter Simple Sequence Repeat
LN	Liquid Nitrogen
MAI	Methylation Analysis Inference
MET	Methyltransferase
metAFLP	methylation-sensitive Amplified Fragment Length Polymorphism
MSAP	Methylation Sensitive Amplified Polymorphism
RAPD	Random Amplified Polymorphic DNA
RdDM	RNA-directed DNA methylation
ROS	Reactive Oxygen Species
SSR	Simple Sequence Repeat
TLC	Thin-layer chromatography
UPGMA	Unweighted pair group method with arithmetic mean
wc.	water content
